# Understanding the nature of association between anxiety phenotypes and anorexia nervosa: a triangulation approach

**DOI:** 10.1186/s12888-020-02883-8

**Published:** 2020-10-07

**Authors:** E. Caitlin Lloyd, Hannah M. Sallis, Bas Verplanken, Anne M. Haase, Marcus R. Munafò

**Affiliations:** 1 Centre for Exercise, Nutrition and Health Sciences, School of Policy Studies, University of Bristol, Bristol, UK; 2 Department of Psychiatry, Columbia University Irving Medical Center, New York, NY USA; 3 Department of Psychiatry, New York State Psychiatric Institute, New York, NY USA; 4 MRC Integrative Epidemiology Unit at the University of Bristol, Bristol, UK; 5 School of Psychological Science, University of Bristol, Bristol, UK; 6 Population Health Sciences, Bristol Medical School, University of Bristol, Bristol, UK; 7 Department of Psychology, University of Bath, Bath, UK; 8 Faculty of Health, Victoria University of Wellington, Wellington, New Zealand; 9 Public Health Sciences Division, Fred Hutchinson Cancer Research Centre, Seattle, Washington USA; 10 National Institute for Health Research Bristol Biomedical Research Centre at the University Hospitals Bristol NHS Foundation Trust and the University of Bristol, Bristol, UK

**Keywords:** ALSPAC, Anorexia nervosa, Anxiety, Longitudinal, Mendelian randomization, Triangulation

## Abstract

**Background:**

Evidence from observational studies suggests an association between anxiety disorders and anorexia nervosa (AN), but causal inference is complicated by the potential for confounding in these studies. We triangulate evidence across a longitudinal study and a Mendelian randomization (MR) study, to evaluate whether there is support for anxiety disorder phenotypes exerting a causal effect on AN risk.

**Methods:**

Study One assessed longitudinal associations of childhood worry and anxiety disorders with lifetime AN in the Avon Longitudinal Study of Parents and Children cohort. Study Two used two-sample MR to evaluate: causal effects of worry, and genetic liability to anxiety disorders, on AN risk; causal effects of genetic liability to AN on anxiety outcomes; and the causal influence of worry on anxiety disorder development. The independence of effects of worry, relative to depressed affect, on AN and anxiety disorder outcomes, was explored using multivariable MR. Analyses were completed using summary statistics from recent genome-wide association studies.

**Results:**

Study One did not support an association between worry and subsequent AN, but there was strong evidence for anxiety disorders predicting increased risk of AN. Study Two outcomes supported worry causally increasing AN risk, but did not support a causal effect of anxiety disorders on AN development, or of AN on anxiety disorders/worry. Findings also indicated that worry causally influences anxiety disorder development. Multivariable analysis estimates suggested the influence of worry on both AN and anxiety disorders was independent of depressed affect.

**Conclusions:**

Overall our results provide mixed evidence regarding the causal role of anxiety exposures in AN aetiology. The inconsistency between outcomes of Studies One and Two may be explained by limitations surrounding worry assessment in Study One, confounding of the anxiety disorder and AN association in observational research, and low power in MR analyses probing causal effects of genetic liability to anxiety disorders. The evidence for worry acting as a causal risk factor for anxiety disorders and AN supports targeting worry for prevention of both outcomes. Further research should clarify how a tendency to worry translates into AN risk, and whether anxiety disorder pathology exerts any causal effect on AN.

## Background

Anorexia nervosa (AN) is a serious eating disorder characterised by persistent restriction of caloric intake and fear of weight gain in the context of a low body weight [1]. The lifetime prevalence rate of AN is estimated to be as great as 4% in women [2]. The disorder has a range of lasting physical health complications, and one of the highest mortality rates of all psychiatric illness [3], yet no single treatment or set of treatments is consistently successful [4].

Despite considerable recent research into AN, with respect to a range of possible causal mechanisms (e.g., genetic, neural, psychological and personality factors), the aetiology remains largely unknown. A number of models of illness propose a causal role of anxiety that does not surround eating and weight gain (i.e., anxiety not explained by a diagnosis of AN) in the development of AN. In particular, it is suggested that for those who develop AN, dietary restriction reduces anxiety, making restrictive eating a valuable coping mechanism, to encourage its continuation [5–8]. Empirical research findings provide some support for such models. Anxiety disorder prevalence is elevated in AN populations, as compared to the general population [9, 10], and retrospective studies report anxiety disorder pathology to precede the onset of AN [9, 11]. The small collection of prospective research provides mixed support for associations between specific anxiety disorder diagnoses and AN development [12–14]. The largest and most comprehensive longitudinal study to date was completed using Danish national registry data [15]. This study reported certain anxiety disorders (generalised anxiety disorder and social phobia) to predict increased risk of future AN onset, but associations did not survive adjustment for all other anxiety/stress disorders (including OCD). Presence of any anxiety/stress disorder was robustly associated with greater risk of subsequent AN, though effects were stronger in men and largely driven by OCD/social phobia. Outcomes potentially reflect that while specific anxiety disorder diagnoses generally cannot explain AN onset beyond anxious pathology that exists across the anxiety disorders, the latter indicates elevated risk of future AN diagnosis. This conclusion is consistent with those of smaller retrospective studies that have found greater general childhood anxiety (i.e. not specific to any given disorder) in individuals who later developed AN (for review see [12, 16]).

Longitudinal studies in which the exposure is measured prior to the outcome are more robust to bias resulting from reverse causation compared with cross-sectional, and retrospective case-control, studies. However, all observational research is vulnerable to bias due to confounding from unmeasured, or inadequately measured, factors [17]. The potential for shared causal risk factors to explain associations between anxiety disorders and AN means that conclusions regarding the causal effects of anxiety disorders on AN cannot be based on findings of longitudinal studies, and the yielded temporal associations, alone. As an example, normative concerns of a given developmental period may interact with vulnerability factors to produce psychiatric pathology. Weight concerns emerging later in the course of development relative to other types of concerns (and in particular, those more typical of earlier childhood) could explain AN occurring after anxiety disorders, rather than reflecting causal effects of anxiety disorders on AN.

Triangulating, or integrating, findings across longitudinal studies with those of alternative design that are subject to different potential biases can strengthen causal inferences [18]. As such, we aimed to compare findings across two studies using different methods to probe associations between anxiety and AN [19]. The precise exposures of interest were worry and anxiety disorders. Worry is defined as a negatively valenced and uncontrollable repetitive thought process that is typically future oriented, and intended to resolve an issue with possible negative outcomes [20]. Worry is a transdiagnostic and cognitive component of anxiety disorders, which comprise a broader collection of cognitive and physical symptoms [21, 22].

Our first study is a longitudinal cohort study that uses data from the Avon Longitudinal Study of Parents and Children (ALSPAC) to determine whether worry, and anxiety disorder presence, at age 10 predict lifetime AN by age 24. The second study employs a two-sample Mendelian randomization (MR) approach [17, 23] to determine whether there is evidence for worry and anxiety disorders causally influencing AN risk. Causal effects in the reverse direction were also explored in Study Two, to further inform the nature of association between anxiety and AN reported in observational studies.

**Fig. 1:**
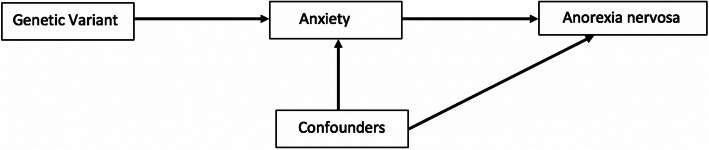
Mendelian randomization analysis

MR (described comprehensively by Davies and colleagues [24]) uses genetic variants associated with an exposure of interest (here, worry and anxiety disorders) as instruments for examining the influence of an exposure on an outcome (Fig. 1; [17]). MR formally assesses the effect of genetic liability to an exposure to provide evidence for causal effects that is, in principle, subject to minimal bias by confounding (including reverse causation) that complicates interpretation of observational research. Converging evidence for an association between anxiety and AN across Studies One and Two would thus provide a stronger basis for causal inference.

Notably, a common genetic liability for various psychiatric disorders (including anxiety disorders and AN) is supported by genome-wide association study data (e.g. [25, 26]), as well as sibling studies (e.g. [27]), and has been labelled the p-factor. Genetic correlations between anxiety disorders (and worry) and AN in particular have also been reported [28–30]. MR is able to further our understanding of these relationships, by informing the mechanisms underlying established genetic associations.

Since worry has been implicated in the development of anxiety disorders (e.g. [22, 31]), we also assess the causal influence of worry on anxiety disorders under a MR framework in Study Two. To assess the specificity of effects of worry, we compare these to effects of depressed affect, and use a multivariable design to assess the independent influence of worry and depressed affect on both anxiety disorders and AN. As a consequence, Study Two offers the potential to highlight particular shared causal risk factors for anxiety disorders and AN that could confound associations in studies of observational design [32]. Outcomes also inform whether the influence of worry is specific, or reflects the operation of negative affect more broadly.

## Study one

### Methods

#### Data sources

The Avon Longitudinal Study of Parents and Children (ALSPAC; [33–35]) is a longitudinal population cohort study. Initially, 14,541 mothers living in Avon, UK, whose expected delivery dates were between 1st April 1991 to 31st December 1992 were recruited. Further eligible mothers have since been recruited, and the total sample comprises 15,247 pregnancies, 14,973 live births, and 14,899 children alive at 1 year. The ALSPAC study website provides details of all available data, through a fully searchable data dictionary and variable search tool (for more information, see: http://www.bristol.ac.uk/alspac/researchers/our-data/). Ethics approval for the study was obtained from the ALSPAC Ethics and Law Committee and the Local Research Ethics Committees.

The present study includes data from all consenting participants alive at 1 year (*n* = 14,882). Demographic information for participants of the current study is shown in Table 1.

**Table 1:** Characteristics of Participants in Study One

Demographic Variable	Frequencies
	*N* (%)
*Sex*	
Male	7601 (51·08)
Female	7280 (48·92)
*Social economic status*	
Manual	2808 (18·87)
Non-manual	9398 (63·15)
Missing	2676 (17·98)
*Ethnicity*	
Non-white	609 (4·09)
White	11,468 (77·06)
Missing	2805 (18·85)
*Mother Parity*	
Primipari	5770 (38·77)
Multipari	7154 (48·07)
Missing	1958 (13·16)

Lifetime AN at age 24 was evaluated by determining, at four data collection waves (when participants were aged 14, 16, 18 and 24 years), whether participants met DSM-5 diagnostic criteria for AN, based on previously defined thresholds (see Micali et al., 2015) outlined in Table 2. Diagnoses were collapsed across the four time-points; if at any time-point a participant met criteria for AN they were considered an AN case, and must have not met criteria at each time-point to be considered a healthy control (or without AN pathology). Participants were not included if data regarding AN diagnosis was missing at one of the time-points and they did not meet criteria for AN otherwise. See the Online Resource for details of AN symptom assessment.

**Table 2:** Criteria Used to Derive Anorexia Nervosa Diagnoses at Each Wave in ALSPAC Sample

Age	Weight criteria	Child report	Parent report
14	Underweight	Self-reported weight/shape concern OR engaged in fasting for weight loss or to avoid weight gain at least monthly OR engaged in excessive exercise	Presence of fear of weight gain AND fat avoidance in the 3 months prior to assessment
16	Underweight	Engaged in fasting for weight loss or to avoid weight gain at least monthly OR engaged in excessive exercise	Presence of fear of weight gain AND fat avoidance in the 3 months prior to assessment
18	Underweight	Self-reported weight/shape concern OR engaged in fasting for weight loss or to avoid weight gain at least monthly OR engaged in excessive exercise	N/A
24	Underweight	Self-reported weight/shape concern OR engaged in fasting for weight loss or to avoid weight gain at least monthly OR engaged in excessive exercise	N/A

Underweight at ages 14–18 was determined using gender specific norms from UK reference data, and corresponded to WHO grade 1 thinness [36]. At age 24 underweight was defined as BMI < 18.5

Anxiety exposures were assessed when children were aged 10 using the parent-report Development and Wellbeing Assessment (DAWBA; [37]), administered to mothers. The DAWBA is a structured interview that generates psychiatric diagnoses for children and adolescents based on ICD-10 [38] and DSM-IV [39] criteria. Worry was assessed with the question ‘Does your child worry?’, with possible response options ‘yes’ or ‘no’, providing a binary variable used in the current investigation. Presence of generalized anxiety disorder, separation anxiety disorder, social phobia, and specific phobia was assessed. Computer algorithms assigned children to DAWBA bands indicating the likelihood of meeting DSM-IV criteria for each anxiety disorder. Children in the top two bands were at least 50% likely to have the anxiety disorder in question and assigned a diagnosis; these diagnoses broadly align with clinician judgements [40]. From assessment of the four anxiety disorders, a binary anxiety disorder variable was derived, indicating whether participants met criteria for any anxiety disorder at age 10. The tetrachoric correlation between the worry and anxiety disorder variables was estimated as 0.45.

**Fig. 2:**
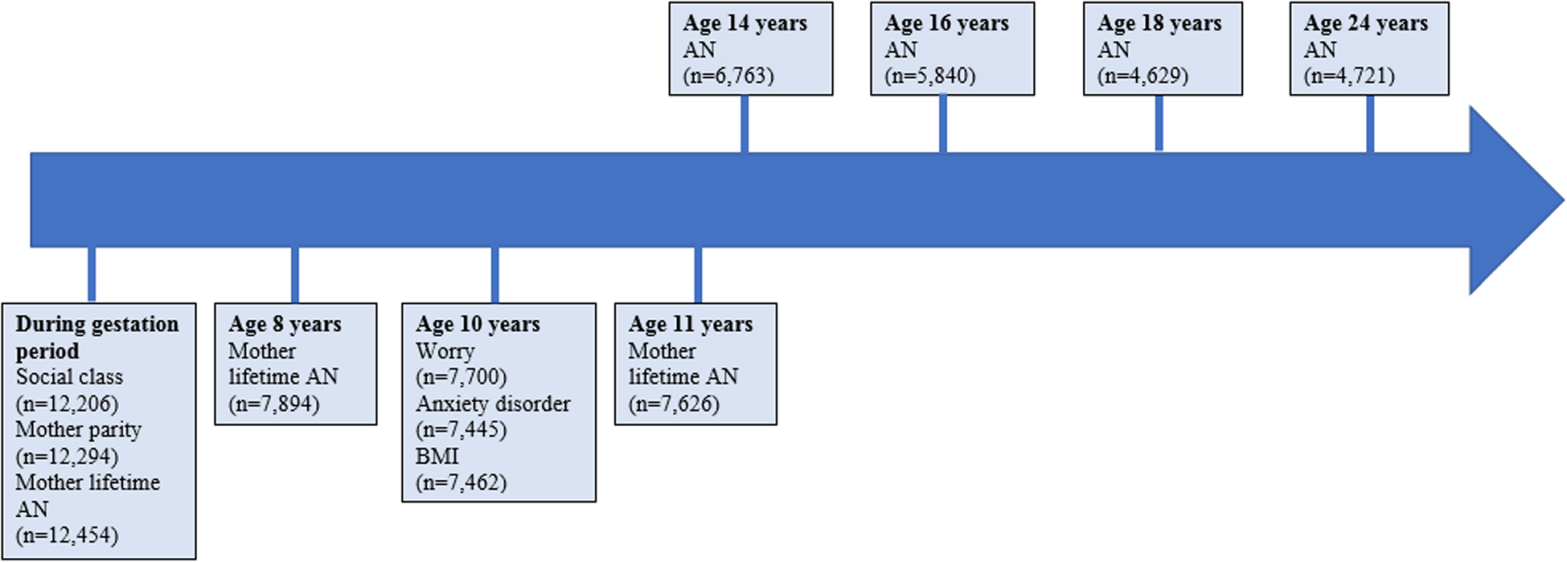
Timeline of data collection for Study One

Plausible confounders of the association between anxiety exposures and AN were identified from the existing literature, and included in statistical models. These were sex, mother lifetime AN, and body mass index (BMI) z-score at baseline (age 10). Symptoms and diagnoses of both anxiety disorders and AN are elevated amongst female adolescents [41, 42], and maternal AN has been associated with child psychopathology [43, 44]. Childhood BMI has been linked to AN, and found to predict both increased and decreased risk [45, 46]; elevated BMI has also been implicated in adolescent anxiety development (e.g. [47]). Three strong predictors of missing data in ALSPAC, used in previous investigations probing associations between eating disorders and other psychiatric outcomes (e.g. [48, 49]), were also included as covariates. This was to minimise the risk of non-random missingness, and subsequent bias in the estimate of association, particularly for analyses without imputed data. The predictors of missingness were: mother age at delivery, socio-economic status (a binary variable based on occupations of both parents), and mother parity (a binary indicator of whether mothers had previous viable pregnancies). Covariate values were determined from questionnaire data, apart from the BMI variable, which was derived from clinic-assessed height and weight, child gender, and UK reference data [50]. Figure 2 shows the data collection process for Study One.

### Statistical analysis

Statistical analyses were completed using Stata v15 [51]. Binary logistic regression models (unadjusted, and adjusted for covariates) assessed longitudinal associations between anxiety phenotypes and AN. Models were subsequently adjusted for the other anxiety exposure (i.e., anxiety disorder presence or worry), to assess the unique variance in lifetime AN explained by worry and anxiety disorders. Given the ALSPAC sample includes related individuals, variance robust standard errors were calculated.

In primary analyses all missing data were imputed using a multiple imputation by chained equation (MICE) approach, which assumes data are missing at random. The imputation model included all analytical model variables. The models also included as auxiliary variables those used to derive AN diagnoses at each wave, to improve prediction. Additional predictors of missingness were not included in the imputation model given analytical models incorporated three such predictors. 100 datasets were imputed.

Whilst a substantial proportion of data was imputed, simulation studies have demonstrated that under these conditions data imputation is able to reduce bias compared to complete case approaches, and achieves the desired gains in efficiency [52]. Further, our imputation model produced data that was similar to that observed, suggesting its appropriate specification (Online Resource, Table S1). Complete case and maximum available data analyses were undertaken for comparison with outcomes of the primary imputed data analyses, and we present outcomes of all models.

## Results

In unadjusted analyses, worry at age 10 was associated with increased risk of AN by age 24, however the statistical evidence provided modest support for the association, with plausible effects (i.e. those within confidence intervals) being both positive and negative in direction (OR = 1.60, 95% CI: 0.93 to 2.77, *p* = 0.09). Furthermore, the association was attenuated towards the null when adjusting for potential confounders, with wide confidence intervals around the estimate resulting in little evidence for an association (OR = 1.41, 95% CI: 0.78 to 2.56, *p* = 0.26). When anxiety disorders were added to the model the magnitude of association was further reduced (OR = 1.34, 95% CI: 0.74 to 2.45, *p* = 0.33).

In unadjusted analyses there was statistical evidence for an association between anxiety disorders and AN, both in terms of the effect estimate and the corresponding confidence intervals, indicating that individuals meeting anxiety disorder criteria at age 10 were more likely to develop AN by age 24 (OR = 2.85, 95% CI: 1.22 to 6.63, *p* = 0.02). In analyses adjusted for potential confounders the results were consistent (OR = 3.12, 95% CI: 1.13 to 8.64, *p* = 0.03). Adding worry to the model also did not alter the results substantially (OR = 2·87, 95% CI: 1.05 to 7.87, *p* = 0.04).

Though less precise, point estimates of associations in complete case and maximum available data analyses were consistent with those of imputed data analyses. Further, confidence intervals overlapped, and the pattern of results was similar, across all three analyses. Full results are displayed in Table 3.

**Table 3:** Estimates of Multiple Logistic Regression Analyses of Lifetime AN at Age 24 on Anxiety Phenotypes

Imputed data analyses				
	N	Variable	OR [95% CI]	*P* value
Unadjusted	14,882	Worry	1.60 [0.93, 2.77]	0.09
	14,882	Anxiety disorder	2.85 [1.22, 6.63]	0.02
^a^Adjusted	14,882	Worry	1.41 [0.78, 2.56]	0.26
	14,882	Anxiety disorder	3.12 [1.13, 8.61]	0.03
^b^Maximally adjusted	14,882	Worry	1.34 [0.74, 2.45]	0.33
	14,882	Anxiety disorder	2.87 [1.05, 7.87]	0.04
**Complete case analyses**				
	N	Variable	OR [95% CI]	*P* value
Unadjusted	1977	Worry	1.76 [0.94, 3.26]	0.08
	1977	Anxiety disorder	3.62 [1.07, 12.23]	0.04
^a^Adjusted	1977	Worry	1.55 [0.82, 2.94]	0.18
	1977	Anxiety disorder	2.97 [0.69, 12.8]	0.14
^b^Maximally adjusted	1977	Worry	1.49 [0.78, 2.84]	0.23
	1977	Anxiety disorder	2.64 [0.61, 11.47]	0.19
**Maximum available data analyses**				
	N	Variable	OR [95% CI]	*P* value
Unadjusted	2396	Worry	1.87 [1.07, 3.27]	0.03
	2338	Anxiety disorder	2.8 [0.84, 9.31]	0.09
^a^Adjusted	2039	Worry	1.55 [0.82, 2.93]	0.18
	1999	Anxiety disorder	3.00 [0.7, 12.91]	0.14
^b^Maximally adjusted	1977	Worry	1.49 [0.78, 2.84]	0.23
	1977	Anxiety disorder	2.64 [0.61, 11.47]	0.19

^a^Adjusted model covariates: sex, socio-economic status, mother parity, mother AN, child body mass index z-score at baseline (age 10). ^b^ Maximally adjusted models include all covariates and the other anxiety phenotype

## Discussion

Outcomes of Study One do not support a robust association between worry at age 10 and later AN development. In contrast, there was evidence supporting the presence of an anxiety disorder at age 10 predicting increased risk of subsequent AN. This latter finding aligns with outcomes of cross-sectional and retrospective research [10]. The association between *any* anxiety disorder and subsequent AN development has been reported previously [12]. The evidence for longitudinal associations between specific anxiety disorder diagnoses and AN development is not strong [53]. However, prior analyses have tested whether particular anxiety disorder diagnoses explain variation in AN onset over and above the explanatory effects of other anxiety disorders [12, 13], when large unique predictive effects may be absent. Alternatively, methodological limitations could have reduced sensitivity to detect associations in past investigations. For example, some studies (e.g. [12, 14]) did not extend follow-up periods to encompass the entire period in which AN onset is most common (i.e., age 15–19 [54]).

The absence of clear evidence for an association between worry and AN conflicts with findings of cross-sectional studies reporting greater worry in AN as compared to healthy controls (e.g. [55],). The finding is also surprising given worry is a core component of anxiety disorders [21]. Worry was measured coarsely in this study however, and the severity of worry indicated by a positive response could have been low or high, potentially masking associations between more severe levels of worry and AN. It is also possible that worry was less accurately reported by parents as compared to other anxiety disorder symptoms, given its unobservable nature [56]. Limitations with the assessment of worry may have rendered the current investigation more sensitive to associations between anxiety disorders and AN, as compared to worry and AN. Notably, whilst the banding strategy used to assign anxiety disorder diagnoses has previously been found to result in underestimates of disorder prevalence relative to clinician-assigned diagnoses, associations between disorders and risk factors have been preserved [40].

Findings were broadly consistent across analyses with complete case, maximally available, and imputed, data, supporting the reliability and validity of analysis outcomes. Statistical adjustment for plausible confounders minimised the risk of biased estimates. However, it is a limitation that disordered cognition and behaviour surrounding eating and weight gain at baseline was not included as a covariate, since this information was not captured in ALSPAC. A final limitation is the single point in time assessment of association between worry/anxiety disorders and AN (with AN diagnosis collapsed across multiple time-points). Although this approach reduced the possibility of reverse causal effects (AN onset prior to age 10 being rare [57]), and avoided problems with sparse data (owing to the rarity of AN at each measurement point), a repeated measures design would allow for capturing more proximal effects.

## Study two

### Methods

#### Data sources

Details of the GWAS data used in the MR study are provided in Table 4. Worry and depressed affect were measured by the Eysenck Personality Questionnaire-Revised Short Form [59] neuroticism subscale (detailed in the Online Resource). Four items assessed worry, and four separate items assessed depressed affect. Derivation of the separate worry and depressed affect factors from the neuroticism scale is empirically supported [60, 61]. The number of ‘Yes’ responses to worry items was summed to derive a quantitative worry phenotype, and only individuals who responded with ‘Yes’ or ‘No’ (deemed valid responses) to all items of a given cluster were included in the GWAS [61]. The same procedure was undertaken to determine a quantitative score, and assess genetic associations with this score, for depressed affect. The anxiety disorder case-control phenotype reflects the presence of five core anxiety disorders (generalized anxiety disorder, panic disorder, social phobia, agoraphobia, specific phobia [58]). The anxiety disorder GWAS data comprised results of a meta-analysis of genome-wide association scans from three large cohorts. The AN phenotype was binary, indicating a diagnosis of lifetime AN, or eating disorder not otherwise specified AN subtype [28]. Participants gave informed consent for study participation and data sharing, as described in articles detailing original GWAS for each phenotype.

**Table 4:** Characteristics of GWAS of Mendelian Randomization Analyses in Study Two

Phenotype	Study	Resource	N genome-wide significant SNPs^**a**^	Total Sample size (N case, N control)	Population	Estimated SNP heritability	Data Source
Worry	Nagel et al. 2018 [29]	UK Biobank	60	348,219	European	9.1%	https://ctg.cncr.nl/software/summary_statistics
Depressed Affect	Nagel et al., 2018 [29]	UK Biobank	60	357,957	European	8.9%	https://ctg.cncr.nl/software/summary_statistics
Anxiety Disorder	Purves et al. 2019 [58]	UK Biobank, ANGST, iPSYCH	2	114,019 (31,977 cases, 82,114 controls)	European	Not reported	https://drive.google.com/drive/folders/1fguHvz7l2G45sbMI9h_veQun4aXNTy1v
Anorexia Nervosa	Watson et al. 2019 [28]	PGC	8	72,517 (16,992 cases 55,525 controls)	European	17.0%	https://www.med.unc.edu/pgc/results-and-downloads/ed./

*ANGST* Anxiety Neuro Genetics STudy; *PGC* Psychiatric Genetics Consortium

^a^ Independent genome-wide significant SNPs identified using LD threshold of R^2^ < 0.001, and distance threshold of > 10,000 kb, based on 1000 genomes reference panel

#### Genetic instrument selection

Genetic instruments for each exposure were identified from relevant GWAS summary statistics (Table 4). A significance threshold of 5 × 10^− 8^ was used to select independent single nucleotide polymorphisms (SNPs) robustly associated with each exposure. To ensure independence, SNPs were clumped using a threshold of linkage disequilibrium r^2^ = 0.001, and a distance of 10,000 kb. Palindromic SNPs were replaced with proxy variants in linkage disequilibrium (r^2^ > 0.80) with original variants. SNPs that were missing in the outcome GWAS were also replaced with proxy variants, for estimation of both SNP-exposure, and SNP-outcome associations. Where only one or two SNPs were identified as eligible instruments, we ran an additional sensitivity analysis using a significance threshold of 5 × 10^− 6^ for instrument identification.

### Statistical analyses

MR analyses were implemented in R [62] using the TwoSampleMR package of MR-Base [63], the gsmr package [64], and locally downloaded GWAS data [28, 29, 58, 65].

#### Univariable analyses

For single SNP instruments the Wald ratio (ratio of coefficients) method was used to estimate the causal effect. Our primary analysis comprised the inverse variance weighted (IVW) analysis, in which Wald ratio estimates across different SNPs were combined using a weighted formula. Here, the contribution of each SNP estimate is inversely proportionate to the variance of the SNP-outcome association.

When there were more than two SNP instruments, multiple sensitivity analyses were completed to determine whether inferences arising from the IVW estimate were valid. MR assumes associations between genetic instruments and the outcome are fully mediated by the exposure, or that there is no unbalanced horizontal pleiotropy [66]. Five multiple instrument analyses more robust to this assumption were completed. Weighted median and weighted mode analyses, which provide consistent causal estimates when a proportion of genetic instruments are invalid [66, 67], were conducted. Generalized Summary-data-based Mendelian randomization (GSMR [68]), which integrates individual SNP estimates in a manner that accounts for variance in the SNP-exposure, as well as the SNP-outcome, association was completed. The HEIDI-outlier method [68] was implemented to detect and remove SNPs meeting criteria for suspected pleiotropic effects in the GSMR analysis, to provide a pleiotropy-corrected estimate of causal association. Finally, two methods that provide an estimate of pleiotropic effects biasing the IVW estimate, as well as a correction for these effects, were completed. These were MR Egger [66], and the Mendelian randomization pleiotropy residual sum and outlier (MR-PRESSO [69]) test. A comprehensive overview of the MR methods implemented has been provided elsewhere [24, 70]. Though we focus on outcomes of the primary IVW analysis, estimates from all sensitivity analyses are presented. The strongest inferences may be drawn when there is convergence (in terms of the direction/magnitude of the point estimate, and range of plausible effects indicated by confidence intervals) across the different methods.

Consistency across the independent SNP estimates that are combined in the IVW analysis also provides strong support for the validity of conclusions arising from the latter [71]. Cochrane’s Q and I^2^ statistics indexed statistical heterogeneity across estimates combined in the IVW analysis. When substantial heterogeneity was detected, leave-one-out analyses were completed: the IVW analysis was completed leaving out one SNP each time, enabling detection of variants having an undue influence on results. Rucker’s Q (Q’), which estimates heterogeneity of SNP estimates while allowing for pleiotropic effects (i.e., for use in MR Egger analyses [72]), was also calculated. Comparing Cochrane’s Q with Rucker’s Q further informs whether MR Egger or IVW models provide a better fit to the data. A larger value of Q compared to Q’, combined with evidence of pleiotropy, would support the MR Egger model [72].

To ensure inferences from MR analyses were directionally accurate, where causal effects were indicated, Steiger filtering was performed [73]. The variance in exposure and outcome explained by the instrument was estimated for each SNP. Where the association between genetic instrument and exposure is stronger than corresponding associations between the same instrument and outcome, a direction of causal effect from exposure to outcome is supported. MR analyses were replicated using the subsample of (filtered) variants meeting this criterion, with consistent results from original and filtered analyses lending support to the validity of the former.

Since estimates from analyses assessing causal effects of genetic liability to binary anxiety disorder and AN exposures do not have a clear interpretation, they were transformed to the liability scale using previously derived formula [74]. These estimates (available in the Supplementary Tables) reflect increases in the outcome per standard deviation increase in exposure liability. The effect sizes we would be able to detect at 80% power and with alpha set to 5% were calculated using the Shiny R application ‘Rmd’ [75], and are also presented in the Supplementary Tables.

**Fig. 3:**
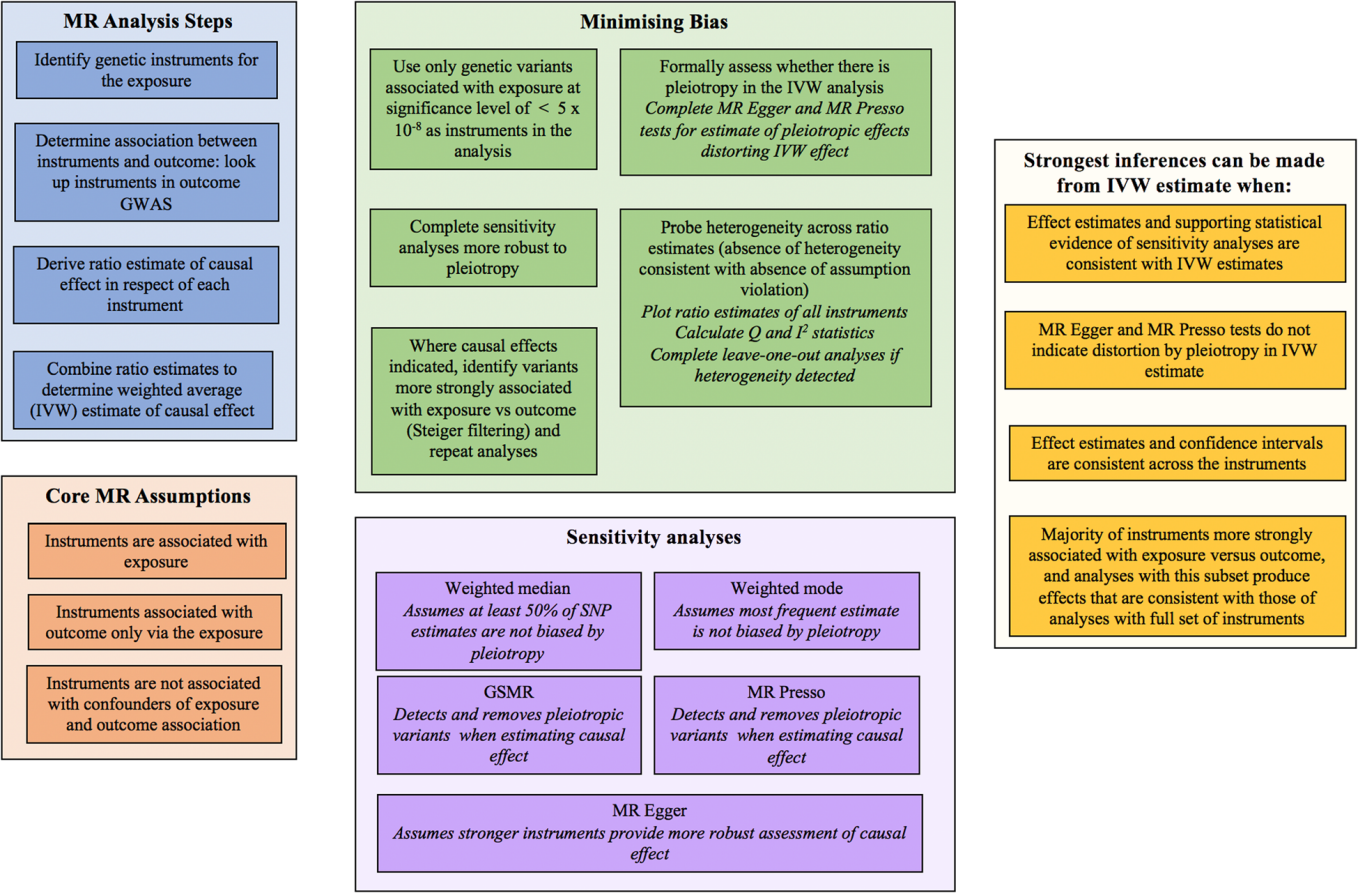
MR methods for Study Two

The steps for completing MR analyses, and evaluating outcomes, are outlined in Fig. 3.

#### Multivariable analyses

Multivariable analyses evaluate the influence of a given exposure that is independent of effects of other (related) exposures, or effects of one trait adjusted for others. The approach allows for determining whether observed causal effects largely operate through other traits (i.e. pleiotropy), or reflect the influence of a less specific phenotype. We assessed the independent effects of genetically-predicted worry and depressed affect (reported to be correlated [29]) on both anxiety disorders and AN, using a multivariable approach.

Our analyses included independent instruments associated with at least one of the worry and depressed affect exposures at the 5 × 10^− 8^ threshold, and that were available in the outcome GWAS. There were 89 variants in the analysis assessing causal effects on AN, and 91 in the analysis assessing causal effects on anxiety disorders. SNP-outcome association estimates were regressed onto SNP-exposure association estimates for both worry and depressed affect, at the same time, with regression weights inversely proportionate to the variance of the SNP-outcome association.

Multivariable MR Egger analyses [76] were completed to determine the robustness of multivariable IVW estimates, and in particular to inform whether pleiotropy was likely to be introducing bias into the IVW estimates. As with univariable MR Egger, the multivariable extension provides an estimate of unmeasured pleiotropy (unaccounted for by inclusion of additional exposures), as well as pleiotropy-corrected estimates of causal effect. SNP estimates were oriented so that the effect allele was the risk-increasing variant with respect to the worry exposure of primary interest, as per existing recommendations [76].

## Results

Figure 4 provides estimates resulting from primary IVW analyses, and all sensitivity analyses, for univariable and multivariable tests. For Wald ratio estimates of instrumental SNPs in each univariable analysis, see the Online Resource (Figures S2-S9).

**Fig. 4:**
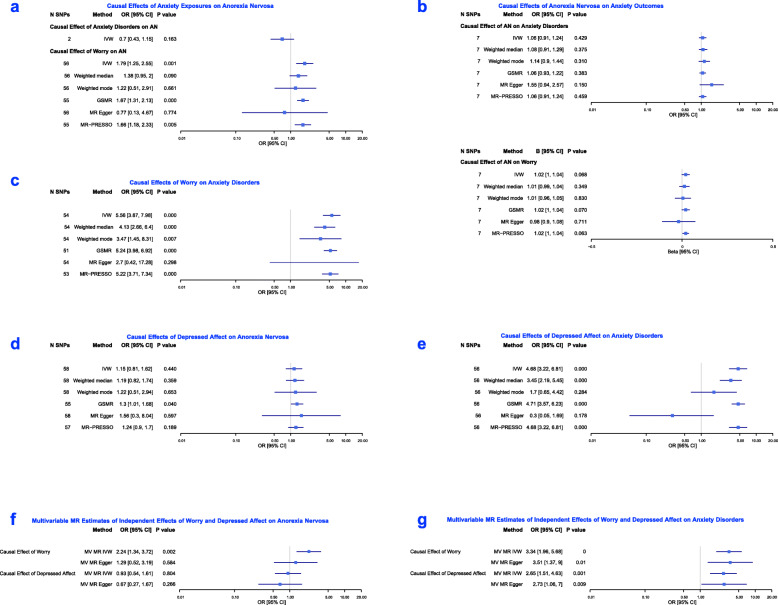
Results of MR analyses of Study Two

### Univariable analyses

#### Effects of anxiety exposures on AN

The primary IVW analysis that included 2 SNPs indicated a negative effect of genetic liability to anxiety disorders on AN risk. However, the confidence intervals were wide, such that there was no strong evidence to support an association. Findings from analyses including additional independent SNPs (less strongly associated with the exposure) were consistent (Online Resource, Figure S1).

The IVW effect estimate and surrounding confidence intervals provided strong evidence that worry increases AN risk (OR = 1.79, 95% CI: 1.25 to 2.55, *p* = 0.001). Estimates of all sensitivity analyses were directionally consistent (other than that of the MR Egger estimate), and corresponding confidence intervals overlapped with those resulting from the IVW analysis. The precision of estimates was such that strong statistical evidence for the association was provided by MR-PRESSO and GSMR analysis outcomes. For all estimates see Fig. 4a.

#### Effects of AN on anxiety phenotypes

A reverse direction of association, from AN to the anxiety phenotypes, was not strongly supported (Fig. 4b). Estimates of effect were close to the null, and (for effects of genetic liability to AN on anxiety disorders) imprecise.

#### Effects of worry on anxiety disorders

The primary IVW analysis provided strong evidence for worry causally increasing risk of anxiety disorder development; the point estimate and surrounding confidence intervals indicated large effects (OR = 5.56, 95% CI: 3.87 to 7.98, *p* < 0.001). This association was strongly supported by outcomes of all sensitivity analyses (in terms of resulting point estimates and the range of plausible values indicated by confidence intervals). All estimates are shown in Fig. 4c.

#### Effects of depressed affect

There was no strong evidence for a causal influence of depressed affect on AN development in the primary IVW analysis, with wide confidence intervals surrounding the effect estimate. Outcomes of the IVW analysis did however provide strong support for depressed affect causally increasing risk of anxiety disorder development (OR = 4.68, 95% CI: 3.22 to 6.81, p < 0.001), as did outcomes of weighted median, GSMR and MR-PRESSO tests. Confidence intervals across all sensitivity analyses largely overlapped with each other, and those of the IVW tests (all estimates shown in Fig. 4d and e).

#### Steiger tests

Steiger filtering was applied to tests of the causal influence of worry on AN and anxiety disorders, and of the causal influence of depressed affect on anxiety disorders. Filtered variants comprised the majority of SNPs in the original analysis; estimates from MR analyses including only the filtered set of variants were consistent with those of original analyses (Online Resource, Figures S10-S12).

#### Pleiotropy tests

There was no strong evidence for bias due to horizontal pleiotropy in the IVW estimates across the analyses. The Egger intercept did not support pleiotropy influencing the IVW estimate, other than for the assessment of the causal influence of depressed affect on anxiety disorders. In all analyses, estimates of the GSMR and MR-PRESSO tests that detect and remove pleiotropic variants were consistent with outcomes of IVW analyses. The MR-PRESSO tests of distortion also did not yield strong evidence for pleiotropic effects biasing inferences arising from IVW analyses.

Whilst heterogeneity amongst SNP estimates was indicated in analyses testing the causal influence of worry and depressed affect (on both AN and anxiety disorders), confidence intervals of each SNP estimate overlapped, and leave-one out analyses did not indicate a disproportionate influence of any single SNP. The comparison of Cochrane’s Q with Rucker’s Q did not support MR Egger models providing a superior model fit relative to IVW models in any multi-SNP analysis, aside from that evaluating causal effects of depressed affect on anxiety disorders. For more detail see the Supplementary Tables and Online Resource (Figures S13-S16).

### Multivariable analyses

The multivariable IVW estimate for the causal influence of worry on risk for AN development (that is independent of effects of depressed affect) indicated a positive association, which was estimated with precision, providing strong evidence of an effect (OR = 2.24, 95% CI: 1.34 to 3.72, *p* = 0.002). In contrast, there was no strong evidence to support an independent effect of depressed affect on AN. The multivariable MR Egger estimates were directionally consistent with those of multivariable IVW analyses, although confidence intervals were wide, to mean there was no clear evidence for a causal effect of worry, nor depressed affect.

The multivariable IVW point estimates and confidence intervals provided strong evidence for large causal effects of worry (that were independent of effects of depressed affect) on anxiety disorders (OR = 3.34, 95% CI: 1.96 to 5.68, *p* < 0.001). Multivariable IVW point estimates and confidence intervals also provided strong evidence for causal effects of depressed affect on anxiety disorders (OR = 2.65, 95% CI: 1.51 to 4.63, *p* = 0.001) that were independent of effects of worry. The multivariable MR Egger point estimates were consistent, and were sufficiently precise to provide strong statistical evidence for independent causal effects of both worry and depressed affect on anxiety disorders. For all estimates of multivariable analyses, see Fig. 4 (panels f and g).

Estimates of the intercept term in multivariable MR Egger analyses were close to zero, suggesting an absence of bias due to directional pleiotropy in the multivariable IVW analyses. For further details, see the Supplementary Tables.

## Discussion

Our MR investigations support a causal influence of worry on AN development, but provide no clear evidence for a causal effect of anxiety disorders on AN, nor for any reverse causal effect from AN to anxiety phenotypes (worry and anxiety disorders). The causal influence of worry on anxiety disorders, as well as AN, was estimated, and strong statistical support for this association was observed. Furthermore, there was evidence that worry explained variance in AN and anxiety disorder development independently of another component of negative affect (depressed affect). Such serves to further support the causal effects indicated in univariable analyses, and suggests that the apparent influence of worry is not simply reflective of general negative affect.

The validity of inferences arising from MR estimates rests on several assumptions. We adopted methods to minimise the potential for violating these assumptions in our primary IVW analyses (e.g. implementing significance/independence thresholds for instrument selection), and completed several sensitivity analyses more robust to the pleiotropic effects that serve as the largest threat to valid inference. Across analyses, outcomes of sensitivity analyses produced effect estimates that were broadly consistent with those of primary IVW analyses, both in direction and size, lending support to conclusions drawn from IVW estimates. Furthermore, the range of plausible effect sizes indicated by estimated confidence intervals generally overlapped across the primary and sensitivity analyses. Statistical evidence provided by the sensitivity analyses was not always as strong as that corresponding to IVW estimates, which should be considered when interpreting the results. Equally important to consider however is the fact that a number of these tests (most notably MR Egger and Weighted Mode) have considerably reduced power to detect causal effects relative to the IVW analysis [66, 67]. Further, across the methods that provided estimates of pleiotropy affecting the primary IVW estimate, substantial bias was not indicated, and methods providing a correction for potential pleiotropy did not collectively indicate distortion in the primary IVW test. Although statistical heterogeneity was evident amongst individual SNP estimates in various analyses, the overlapping confidence intervals, combined with absence of any large inconsistencies, further supports the validity of summary estimates. Causal effects indicated in primary analyses were also supported by outcomes of Steiger filtering, and effect estimates of analyses completed with the majority subset of variants more strongly associated with exposure relative to outcome. We do note though that the statistical evidence tended to be weaker as compared to analyses completed with all variants.

Importantly, the evidence in Study Two that worry, but not anxiety disorders, may causally influence AN outcomes is inconsistent with evidence from Study One. This may be due to confounding of the anxiety disorder and AN association in Study One, given that MR is intended to protect against this. The evidence for a causal influence of worry on AN risk in the MR investigation supports the possibility that limitations in worry measurement in Study One clouded a true association between worry and AN. The finding that worry increased risk of anxiety disorder development is consistent with conclusions drawn from a prior MR investigation [29]. It also aligns with outcomes of randomized-controlled trials (RCTs) that have manipulated worry to observe subsequent changes in anxiety symptomatology (e.g. [22]). Collectively the findings suggest worry may act as a shared risk factor of AN and anxiety disorders, and a factor that could confound the association between AN and anxiety disorders in observational studies.

Depressed affect did not show the same associations with AN; there was little evidence for causal effects of depressed affect on AN in univariable or multivariable analyses. Both worry and depressed affect are subcomponents of neuroticism, and the assessed factors were in fact drawn from a broader neuroticism subscale. Neuroticism is proposed as a causal risk factor for multiple psychiatric pathologies [77], and has been associated with anxiety disorders and AN previously (e.g. [78–80]). The findings suggest that worry is the component of neuroticism particularly relevant for AN development, and which may contribute to shared risk for anxiety disorders and AN. This explanation is more compelling in the context of how comparable the worry and depressed affect exposures are. Worry and depressed affect were assessed in the same population, using the same questionnaire format. Both exposures had a similar number of SNP instruments associated with them, with instruments explaining a similar amount of variance in exposure, to mean analyses were similarly powered [81].

In addition to assessing, and taking measures to minimise, bias in effect estimates, we used the largest available GWAS for exposure and outcome in each analysis, to enhance power. Nonetheless, analyses probing causal effects of genetic liability to anxiety disorders and AN were under-powered except for the detection of moderate/large effects (see Supplementary Tables). This results from there being few genetic instruments in the relevant analyses (for overview/explanation see [24]). The small number of variants robustly associated with anxiety disorder and AN phenotypes, which serve as instruments in MR analyses, is presumably due to the relatively small size of the respective GWAS (in particular the low number of cases). In respect of anxiety disorders, heterogeneity amongst cases combined in the GWAS may also have contributed to the low number of variants associated with the phenotype. As such, the absence of evidence for a causal influence of anxiety disorders or AN should not be interpreted as the lack of causal association. Although this does not detract from the causal effects that were observed, it does limit comparability of causal influences between phenotypes.

### General discussion

In Study One, we found that anxiety disorders present at age 10 predict subsequent AN development, but there was no evidence to support a similar association between worry and AN. In Study Two, we did not find evidence to support the association between anxiety disorders and AN being causal (in either direction), but did find evidence that worry may play a causal role in both AN and anxiety disorder development. Study Two also suggested that causal effects of worry on AN were unique, in that the same associations were not observed with respect to a depressed affect exposure. Effects of worry on both AN and anxiety disorders appeared to exist independently of depressed affect, rather than reflecting the influence of negative affect more generally. Triangulating findings across these two studies, each with different strengths, limitations and sources of bias (see Table 5 for an overview), allows for more robust conclusions concerning the nature of association between anxiety exposures and AN [18, 19].

**Table 5:** Assumptions of the Study Designs and Action Taken to Satisfy Them

Observational Study		MR study	
Assumption	Action to satisfy assumption and minimise bias	Assumption	Action to satisfy assumption and minimise bias
Absence of unmeasured or residual confounding	Inclusion of potential confounders in analysis models	Absence of pleiotropic effects that influence outcome	Use of genetic instruments associated with exposure at genome-wide significance level; assessment of pleiotropy in the IVW estimate; comparison of IVW estimate with those of sensitivity analyses more robust to pleiotropic effects; assessment of consistency of instrument effects
Absence of reverse causation	Assessment of anxiety exposures prior to most common period of AN onset	Absence of association between genetic instrument and confounders of exposure-outcome association	Use of genetic instruments associated with exposure at genome-wide significance level; assessment of consistency of instrument effects; use of GWAS completed in European samples only
Missing data does not depend on unobserved data	Imputation of missing data; inclusion of predictors of missingness in imputation and analysis models; comparison of models with imputed, maximum available and complete case data	Robust association between genetic instrument and exposure	Use of genetic instruments associated with exposure at genome-wide significance level

The evidence for a causal influence of worry on both anxiety disorders and AN supports the possibility that anxiety disorders and AN are related due to the two sharing causal risk factors. A recent study probed associations between independent transdiagnostic anxiety disorder factors (measured at age 10) and lifetime AN by age 16, in the same population cohort as that of Study One [16]. In this earlier investigation, a quantitative worry component (derived from a factor analysis, and reflecting worry across multiple domains) predicted AN development, while alternative anxiety disorder components did not. This finding is discrepant with outcomes of our longitudinal analysis. The discordance may be explained by different operationalisations of worry (i.e., the tendency to worry, versus the tendency to worry about multiple different things), and our focus on anxiety disorder diagnoses rather than other transdiagnostic symptoms. Nonetheless, outcomes of the previous study [16] are consistent with the suggestion that worry is the component of anxiety disorders that specifically increases risk of AN, and that contributes to the anxiety disorder and AN association. Studies failing to fully account for the influence of worry when evaluating the association between anxiety disorders and AN may thus obtain inflated effect size estimates that give rise to invalid conclusions. Our first study may have been vulnerable to this, given the relatively crude nature of worry assessment, which likely also limited our ability to detect predictive effects of worry.

In terms of how worry may increase AN risk, perhaps the focus on eating and weight, and even the neurobiological effects of dietary restriction, alleviates worry surrounding topics unrelated to eating and weight in individuals who develop AN. This could encourage continued engagement in AN behavior, and would be consistent with proposals that concerns not explained by AN diagnosis are causal in disorder onset (e.g. [5–8]). Alternatively, it is only when worry becomes directed onto eating and weight that fears of weight gain and severe dietary restriction, or AN pathology, manifests. Certainly, individuals with AN have elevated worry generally, but concern is particularly heightened in relation to eating, weight and shape [82], and this concern is considered a maintaining factor of illness. Here, worry comprises a process that independently contributes to risk of both anxiety disorders and AN, as has been suggested for personality and neuropsychological traits [83–85]. There may exist a cluster of shared risk factors for anxiety disorders and AN that includes worry, and which potentially mediates effects of a broader underlying genetic liability for psychopathology [30, 85, 86]. The specificity of the effects of worry on AN observed in this study is consistent with suggestions that while a genetic psychopathology (or p) factor operates to increase vulnerability to psychiatric disorders generally, it does so via certain traits that are shared to greater or lesser extent between diagnoses (e.g. [26]). This would account for the high co-occurrence of certain disorders [25]. It is also consistent with the observed genetic correlation between anxiety disorders and AN, but suggests the shared genetic risk acts as a common vulnerability, rather than a causal pathway from one disorder to another.

Worry has been targeted in prevention interventions for anxiety disorders, with favourable outcomes further supporting the thinking process as a causal risk factor [22]. Further research, and ideally that of randomised trial design, is required to validate our findings concerning the causal influence of worry on AN development. Existing AN prevention interventions largely do not address non-specific cognitive processes or pathology, tending to focus on reducing disordered eating/weight-associated cognition and behaviour [87, 88]. Future trials might explore whether the addition of modules that address non-specific worry can improve outcomes of existing interventions. These studies should also seek to elucidate the mechanisms by which a tendency to worry influences AN.

It is recognised that conclusions concerning associations between anxiety disorders and AN drawn across the studies have largely been made in light of the limitations of Study One, or observational research, and it is necessary to acknowledge the shortcomings of Study Two. Given low power in the respective analyses, causal effects between anxiety disorders and AN cannot be ruled out on the basis of our findings, and these associations require further assessment when stronger instruments become available. The existence of shared risk factors for anxiety disorders and AN certainly does not preclude causal effects between the two. However, it does mean power in MR studies might be lower than what would be expected on the basis of effect estimates from observational research. Like observational research, MR makes a number of assumptions. Whilst we adopted methods to minimise the risk of violating these assumptions, it remains possible that they were, and that false inferences resulted. It is important to note that although MR minimises bias due to confounding, it is possible for genetic instruments to be associated with confounders of a given exposure-outcome association, which could bias estimates of effect. Consistent findings of the observational and MR study would have provided the strongest evidence for an influence of worry on AN. Nevertheless, confidence in MR findings surrounding the influence of worry is enhanced by alignment between MR and RCT outcomes (at least with respect to the worry and anxiety disorder association). Finally, one limitation that should be considered when drawing conclusions across the two studies is that the study populations differed in age. This could have contributed to the discrepant results of Study One and Study Two, and highlights the importance of exploring age-dependent effects of anxiety exposures on AN development in future work.

## Conclusions

We triangulated findings across a longitudinal cohort study and a MR investigation to inform the nature of association between anxiety phenotypes and AN. While results across studies were not consistent, findings from MR analyses provided support for a causal influence of worry on AN development, highlighting potential utility in addressing worry for AN prevention. Outcomes also supported worry causally influencing anxiety disorder development, and subsequently the potential for shared causal risk factors to inflate estimates of association between anxiety disorders and AN in observational research. The evidence to support a causal influence of anxiety disorders on AN, and of AN on anxiety outcomes, was weak, however interpretation is complicated by low power in the relevant MR analyses. Further exploration of causal effects between anxiety disorders and AN is recommended, and future studies should seek to elucidate mechanisms by which worry may translate into risk for AN and other psychiatric outcomes.

## Supplementary information

**Additional file 1.**

**Additional file 2.**

## Data Availability

The GWAS study data is publicly available and may be downloaded from the websites detailed in Tables 4 and 5. ALSPAC data is not publicly available but proposals to use the data may be made; see the study website for more information (http://www.bristol.ac.uk/alspac/researchers/access/).

## Abbreviations

AN: Anorexia nervosa
ANGST: Anxiety NeuroGenetics Study Consortium
ALSPAC: Avon Longitudinal Study of Parents and Children
GSMR: Generalised Summary-data-based Mendelian randomization
GWAS: Genome wide association study
IVW: Inverse variance weighted
MR: Mendelian randomization
MR-PRESSO: Mendelian randomization pleiotropy residual sum and outlier
OR: Odds ratio
PGC: Psychiatric Genomics Consortium
SNP: Single nucleotide polymorphism
