# Supplementary material for: Understanding the nature of association between anxiety phenotypes and anorexia nervosa: a triangulation approach
Source: BMC Psychiatry. 2020 Oct 7;20:495. doi: 10.1186/s12888-020-02883-8 (PMC7542378; doi:10.1186/s12888-020-02883-8)
Supplement: Supplementary file 2 — Additional file 2. [file 12888_2020_2883_MOESM2_ESM.docx]

**Electronic Supplementary Material**

**Article Title:** Understanding the nature of association between anxiety phenotypes and anorexia nervosa: a triangulation approach

**Journal:** BMC Psychiatry

**Authors:** E Caitlin Lloyd, Hannah M Sallis, Bas Verplanken, Anne M Haase, Marcus R Munafò

**Study One**

**Anorexia nervosa symptom assessment**

To establish the presence of anorexia nervosa (AN) symptoms that informed diagnostic indicators, at each wave participants answered questions surrounding eating and exercise behaviours adapted from those of the Youth Risk Behaviour Surveillance System (1). Fasting was assessed with the question “How often in the past year have you fasted (not eaten for at least a day) to lose weight or avoid gaining weight?”. Excessive exercise was recorded when participants reported exercising for weight-loss or to avoid weight-gain *and* one of the following: exercising despite illness/injury; exercise interfering with other activities; experiencing guilt when missing an exercise session. Body dissatisfaction was probed at wave 14, 18 and 24, using questions of the weight/shape concern scale of the McKnight Risk Factor Questionnaire (2). The number of items administered varied with wave, and body dissatisfaction was recorded as present if the mean response met a previously used threshold (3), which equated to high levels of weight concern. Self-report data at age 24 were collected and managed using REDCap electronic data capture tools hosted at the University of Bristol (4). Parent-reported child AN symptoms were collected using the DAWBA assessment (5) and marked as present when symptoms were severe or extreme. Objective height and weight measurements collected during clinic assessments at each of the waves was used to determine whether participants were underweight.

**Imputed Data**

Table S1: Comparison between observed and imputed data for analysis variables

| **Variable** | **Complete case** | | **Imputed** | |
| --- | --- | --- | --- | --- |
| *Lifetime AN* | Proportion (N) | N | Proportion | N |
| No | 0.97 (74) | 2,634 | 0.96 | 12,248 |
| Yes | 0.03 (2,560) |  | 0.04 |  |
| *Age 10 Anxiety Disorder* |  |  |  |  |
| No | 0.98 (7,282) | 7,445 | 0.98 | 7,437 |
| Yes | 0.02 (163) |  | 0.02 |  |
| *Age 10 Worry* |  |  |  |  |
| No | 0.41 (3,169) | 7,700 | 0.43 | 7,182 |
| Yes | 0.59 (4531) |  | 0.57 |  |
| *Mother Parity* |  |  |  |  |
| Primipari | 0.45 (5,770) | 12,924 | 0.47 | 1,958 |
| Multipari | 0.55 (7,154) |  | 0.53 |  |
| *Social class* |  |  |  |  |
| Manual | 0.23 (2,808) | 12,206 | 0.28 | 2,676 |
| Non-manual | 0.77 (9,398) |  | 0.72 |  |
| *Mother Lifetime AN* |  |  |  |  |
| No | 0.95 (7,397) | 7,759 | 0.95 | 7,123 |
| Yes | 0.05 (362) |  | 0.05 |  |
|  | M (SE) | N | M(SE) | N |
| *BMI z-score at age 10* | 0.32 (0.01) | 7,462 | 0.38 (0.02) | 7,420 |

**Study Two**

**Neuroticism subscale of Eysenck Personality Questionnaire-Revised Short Form (6)**

1. Does your mood often go up and down?
2. Do you ever feel ‘just miserable’ for no reason?
3. Are you an irritable person?
4. Are your feelings easily hurt?
5. Do you often feel ‘fed-up’?
6. Would you call yourself a nervous person?
7. Are you a worrier?
8. Would you call yourself tense or ‘highly strung’?
9. Do you worry too long after an embarrassing experience?
10. Do you suffer from ‘nerves’?
11. Do you often feel lonely?
12. Are you often troubled by feelings of guilt?

Worry subscale = items 6-8, 10.

Depressed affect subscale = items 1,2,5,11.

**Single SNP Estimates in Univariable Analyses**

**
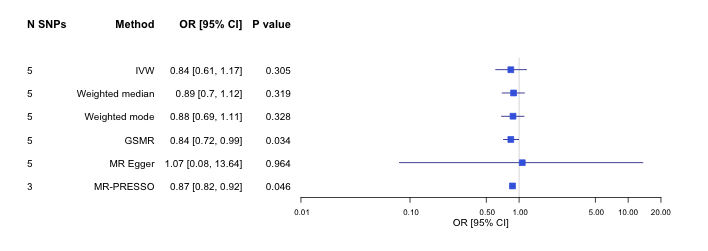
**

Figure S1: Summary Estimates for causal influence of genetic liability to anxiety disorders on AN using reduced threshold for instrument identification

**
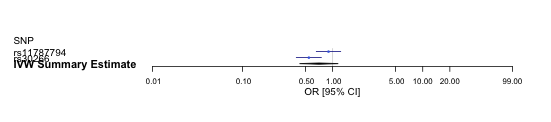
**

Figure S2: Individual SNP MR estimates for causal influence of genetic liability to anxiety disorders on AN

**
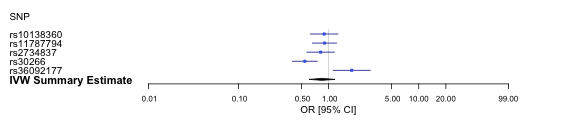
**

Figure S3: Individual SNP MR estimates for causal influence of genetic liability to anxiety disorders on AN using reduced threshold for instrument identification


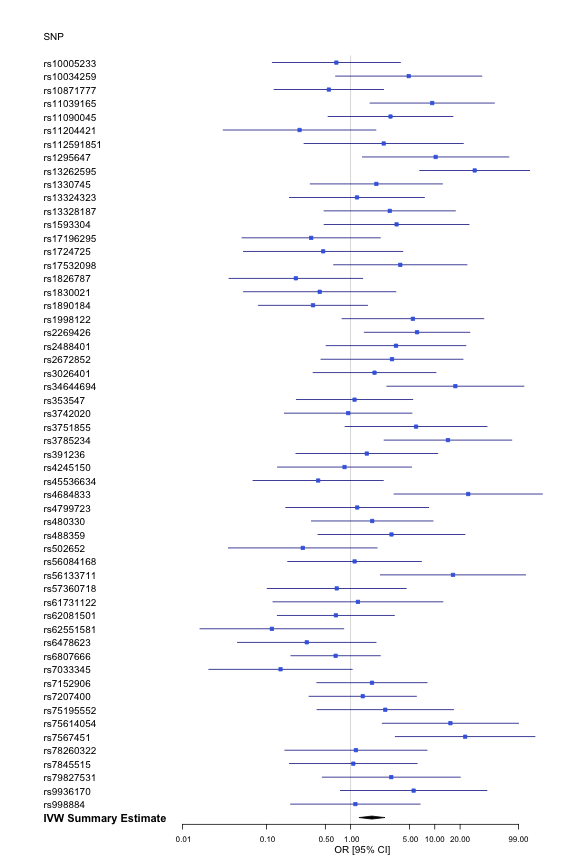


Figure S4: Individual SNP MR estimates for causal influence of worry on AN


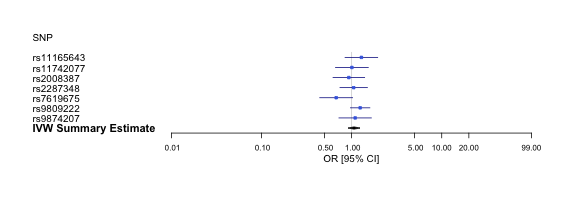


Figure S5: Individual SNP MR estimates for causal influence of AN on Anxiety Disorders


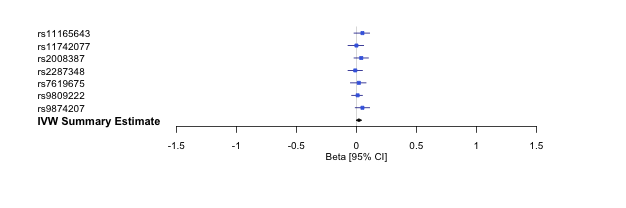


Figure S6: Individual SNP MR estimates for causal influence of AN on Worry


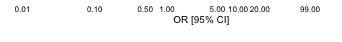

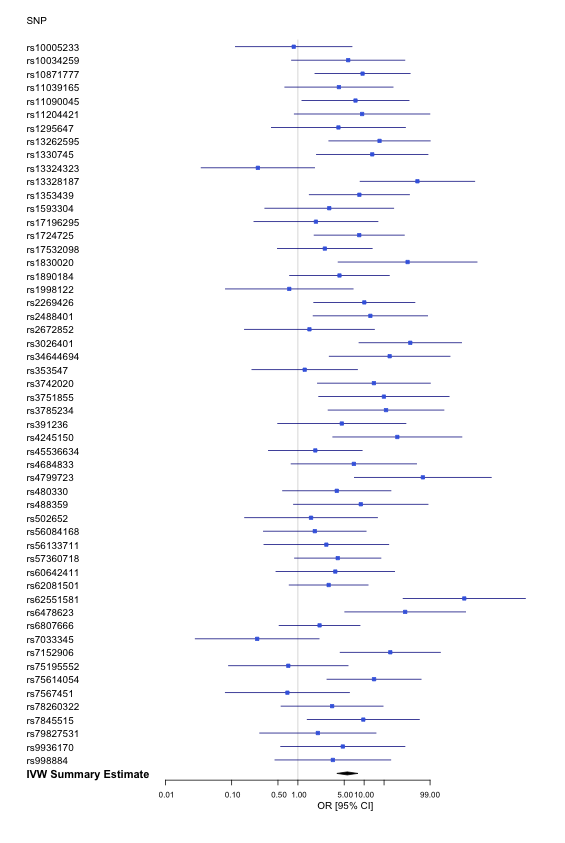


Figure S7: Individual SNP MR estimates for causal influence of Worry on Anxiety Disorders


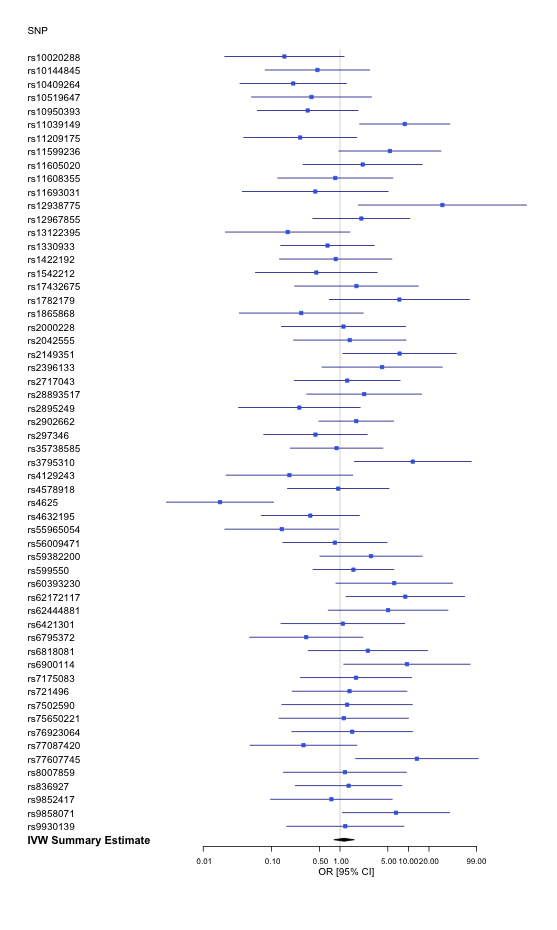


Figure S8: Individual SNP MR estimates for causal influence of Depressed Affect on AN


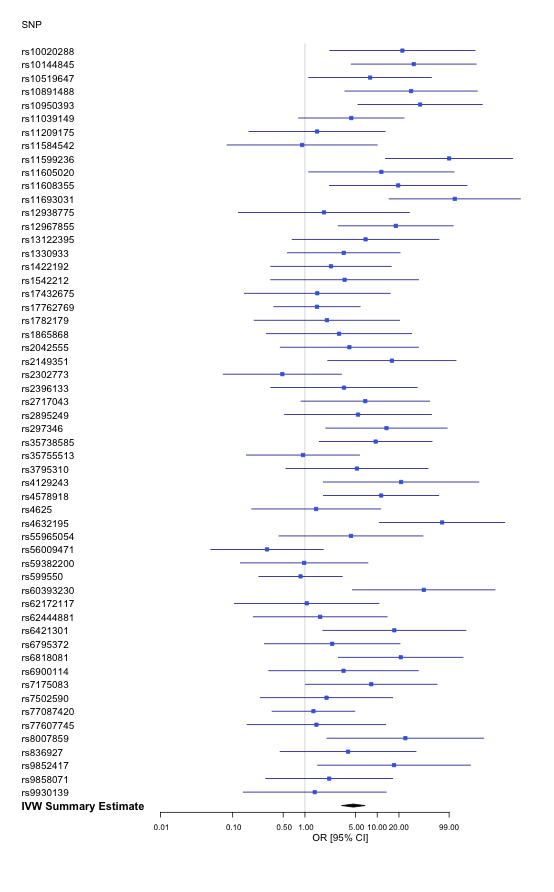


Figure S9: Individual SNP MR estimates for causal influence of Depressed Affect on Anxiety Disorders

**MR Steiger Analyses to Evaluate Direction of Effects**


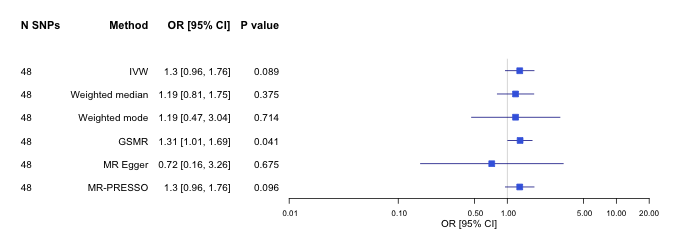


Figure S10: MR analysis to evaluate causal influence of worry on AN using genetic instruments estimated to be more strongly associated with worry as compared to AN


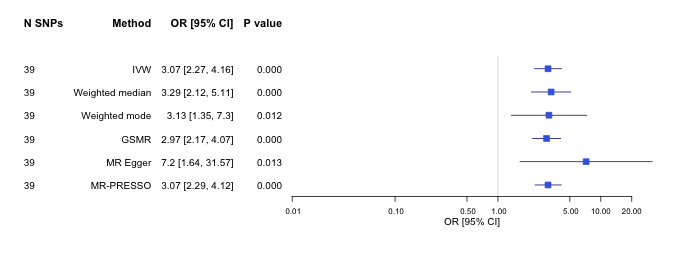


Figure S11: MR analysis to evaluate causal influence of worry on anxiety disorders using genetic instruments estimated to be more strongly associated with worry as compared to anxiety disorders


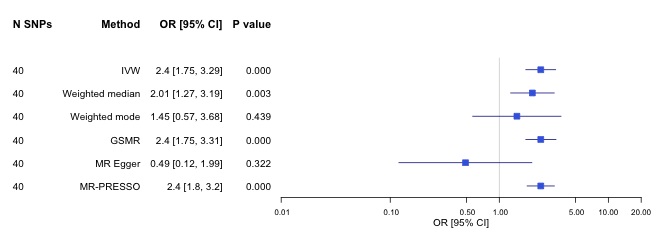


Figure S12: MR analysis to evaluate causal influence of depressed affect on anxiety disorders using genetic instruments estimated to be more strongly associated with depressed affect as compared to anxiety disorders

**Additional Sensitivity Analyses Following Detection of Heterogeneity in MR Estimates**


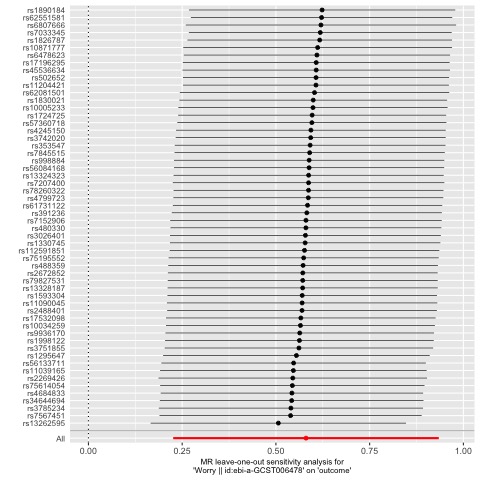


beta

Figure S13: Leave one out analysis for MR IVW estimates of causal effect of worry on AN

Beta [95%CI]

*Additional Sensitivity Analyses following detection of pleiotropy for Study Three*


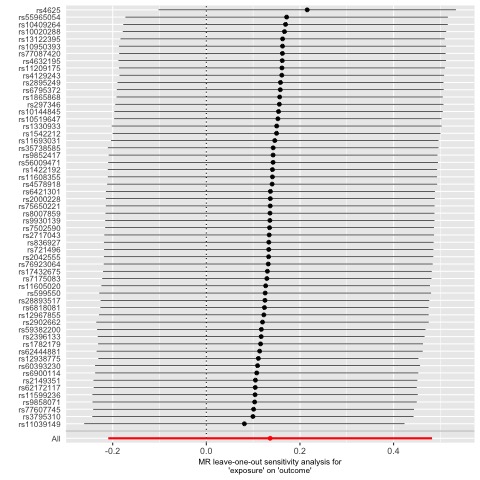


beta

Figure S14: Leave one out analysis for MR IVW estimates of causal effect of depressed affect on AN

**
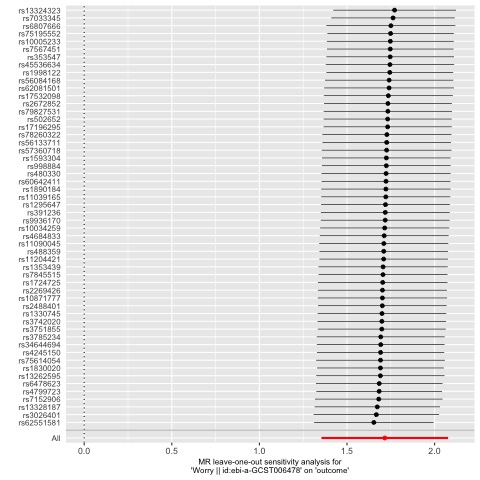
**

beta

Figure S15: Leave one out analysis for MR IVW estimates of causal effect of worry on anxiety disorders

**
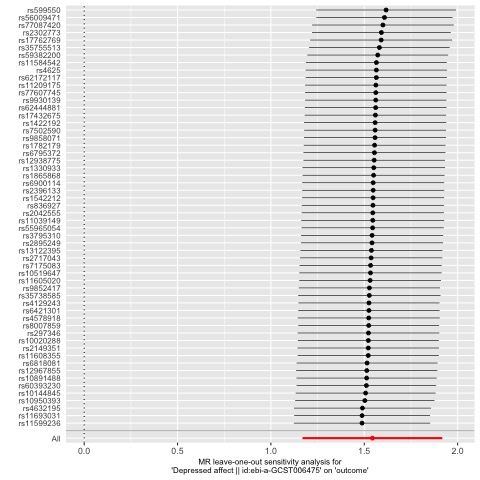
**

beta

Figure S16: Leave one out analysis for MR IVW estimates of causal effect of depressed affect on anxiety disorders

***References***

1. Kann L, Warren CW, Harris WA, Collins JL, Williams BI, Ross JG, et al. Youth risk behavior surveillance--United States, 1995. J Sch Health. 1996;66(10):365-77.

2. Shisslak CM, Renger R, Sharpe T, Crago M, McKnight KM, Gray N, et al. Development and evaluation of the McKnight Risk Factor Survey for assessing potential risk and protective factors for disordered eating in preadolescent and adolescent girls. Int J Eat Disord. 1999;25(2):195-214.

3. Field AE, Camargo CA, Taylor CB, Berkey CS, Roberts SB, Colditz GA. Peer, parent, and media influences on the development of weight concerns and frequent dieting among preadolescent and adolescent girls and boys. Pediatrics. 2001;107(1):54-60.

4. Harris PA, Taylor R, Thielke R, Payne J, Gonzalez N, Conde JG. Research electronic data capture (REDCap)--a metadata-driven methodology and workflow process for providing translational research informatics support. Journal of biomedical informatics. 2009;42(2):377-81.

5. Goodman R, Ford T, Richards H, Gatward R, Meltzer H. The Development and Well-Being Assessment: description and initial validation of an integrated assessment of child and adolescent psychopathology. J Child Psychol Psychiatry. 2000;41(5):645-55.

6. Eysenck SBG, Eysenck HJ, Barrett P. A revised version of the psychoticism scale. Personality and Individual Differences. 1985;6(1):21-9.
